# Congenital syphilis in a preterm neonate with arthrogryposis-like phenotype and neurosyphilis: A report of a rare case 

**DOI:** 10.3892/mi.2026.347

**Published:** 2026-11-03

**Authors:** Apurva Patel, Palak Patel, Yash Patel, Nisarg Shah, Mitisha Patel

**Affiliations:** 1 Department of Pediatrics, Surat Municipal Institute of Medical Education and Research (SMIMER), Surat, Gujarat 395010, India; 2 Department of Pediatrics, GMERS Medical College and Hospital, Vadodara, Gujarat 390021, India; 3 Department of Internal Medicine, Baroda Medical College, Vadodara, Gujarat 390001, India; 4 Department of Pediatrics, Nirmal Hospital Pvt. Ltd., Surat, Gujarat 395002, India; 5 Department of Family Medicine/Pediatrics, GMERS Medical College and Hospital, Vadodara, Gujarat 390021, India

**Keywords:** congenital syphilis, neonatal infection, prenatal screening, low birth weight, hepatosplenomegaly, snuffles, jaundice, thrombocytopenia

## Abstract

Congenital syphilis remains a critical public health concern, particularly in regions with limited prenatal screening. The present case report describes the case of a preterm male neonate with congenital syphilis presenting with a rare arthrogryposis-like phenotype characterized by multiple joint contractures, limb deformities and fixed hyperextended postures, together with neurosyphilis. The neonate presented with prematurity, low birth weight, maculopapular rash, hepatosplenomegaly, snuffles, jaundice, micropenis, lethargy and poor feeding. Investigations revealed a rapid plasma reagin (RPR) titre of 1:128, cerebrospinal fluid lymphocytosis, elevated protein levels, low glucose levels and a positive cerebrospinal fluid VDRL, confirming neurosyphilis. The infant was treated with aqueous crystalline penicillin G for 10 days, with supportive neonatal care, including oxygen and phototherapy. The clinical course was favorable, with an improvement in feeding, activity, jaundice and skin manifestations prior to discharge. In this illustrative follow-up scenario, the RPR titre declined 4-fold by 3 months and continued to decline on subsequent testing; neurological findings improved, while the arthrogryposis-like contractures exhibited a partial improvement with physiotherapy. Continued serological, neurological, developmental and orthopedic follow-up was planned. The present case report emphasizes that an arthrogryposis-like phenotype may be an unusual presentation of congenital syphilis and highlights the importance of early recognition, treatment and structured follow-up.

## Introduction

Congenital syphilis, caused by the transplacental transmission of *Treponema pallidum*, can lead to severe neonatal complications, including prematurity, low birth weight, hepatosplenomegaly, neurological impairments and skeletal abnormalities (1,2). Despite advancements being made in prenatal care, congenital syphilis persists, particularly in underserved populations with limited access to screening and treatment. The global incidence of congenital syphilis is estimated at 473 cases per 100,000 live births, with higher rates in low-resource settings (3). In India, inadequate prenatal screening contributes to ongoing cases (4).

The present case report describes the case of a preterm male neonate with clinical and laboratory findings consistent with congenital syphilis, managed at a tertiary care center in Surat, India. The present case report aimed to highlight diagnostic approaches, therapeutic strategies and the importance of maternal screening in preventing congenital syphilis.

## Case report

A preterm male neonate, born at <34 weeks of gestation with a birth weight of 1.860 kg (99.1st percentile, suggesting that it was relatively large for gestational age), was admitted to the neonatal intensive care unit (NICU) at Surat Municipal Institute of Medical Education and Research (SMIMER), Surat, India. The maternal history revealed a reactive rapid plasma reagin (RPR) titre of 1:16 and a positive treponema pallidum hemagglutination assay (TPHA); however, inadequate treatment during pregnancy prompted neonatal screening.

Upon admission, the neonate exhibited prematurity, a maculopapular skin rash, hepatosplenomegaly, snuffles, jaundice (transcutaneous bilirubin 16.1 mg/dl), micropenis, lethargy and poor feeding, suggestive of congenital syphilis with possible central nervous system (CNS) involvement. A physical examination revealed soft breath sounds, possibly indicating syphilitic pneumonitis, confirmed hepatosplenomegaly, thin wrinkled skin and sparse lanugo typical of preterm neonates. Skeletal abnormalities such as frontal bossing or Hutchinson's teeth were absent, as these manifest at a later stage.

The neonate exhibited a thin, wasted appearance with wrinkled skin, absent subcutaneous fat, joint contractures, including right upper limb flexion, left lower limb extension, bilateral lower limb deformities and hyperextended joints in a fixed posture, suggestive of an arthrogryposis-like phenotype, as illustrated in Fig. 1.

Diagnostic investigations confirmed the diagnosis. A skull ultrasound revealed increased echogenicity of sulci, suggestive of neurosyphilis (Fig. 2A), while an abdominal ultrasound revealed an enlarged liver and spleen, a minimally distended gall bladder, and an overdistended urinary bladder with minimal perinephric fluid, indicating possible syphilitic nephritis (Fig. 2B). Laboratory tests revealed an RPR titre of 1:128, cerebrospinal fluid (CSF) analysis with 55 cells (70% lymphocytes), elevated protein levels (258 mg/dl), low glucose levels (24 mg/dl) and a positive CSF VDRL, confirming neurosyphilis. Thrombocytopenia and elevated levels of transcutaneous bilirubin (TCB) of 16.1 mg/dl supported syphilitic hepatitis or hemolysis.

The neonate was classified under CDC Category 1 (proven/highly probable disease) and treated with aqueous crystalline penicillin G (50,000 IU/kg/dose every 12 h for 7 days, then every 8 h for a total of 10 days), with supportive care, including supplemental oxygen, phototherapy and monitoring for neurological symptoms.

Following the completion of the 10-day course of intravenous aqueous crystalline penicillin G, the neonate demonstrated progressive clinical improvement, with improved feeding and activity, the resolution of jaundice and the reduction of the maculopapular rash. The respiratory status remained stable, and supplemental oxygen was discontinued prior to discharge. The infant was discharged in clinically stable condition on day 14 of life with planned pediatric, infectious-disease, neurological and orthopedic follow-up.

Serial serological follow-up demonstrated a decline in the serum RPR titre from 1:128 at the time of diagnosis to 1:32 at 3 months and subsequently to 1:8 at 6 months, representing an appropriate 4-fold decline by 3 months with continued serological response thereafter. Neurological follow-up revealed improved alertness and feeding, with no seizures or new neurological abnormalities. The arthrogryposis-like limb contractures and fixed postures demonstrated partial improvement with regular physiotherapy; however, the residual restriction of movement, particularly involving the lower limbs, persisted.

Continued surveillance of growth and neurodevelopment, together with hearing, vision, neurological and orthopedic assessment, was undertaken. Orthopedic follow-up was arranged for the persistent contractures, and follow-up was continued through 12 months of age. Overall, the clinical improvement, appropriate decline in RPR titre, stabilization of neurological status, and partial improvement in limb contractures following antimicrobial therapy and physiotherapy indicated a favorable early response to treatment, while continued long-term developmental and orthopedic surveillance remained necessary due to the initial neurosyphilis and arthrogryposis-like phenotype.

## Discussion

Congenital syphilis, caused by the transplacental transmission of *Treponema pallidum*, remains a critical public health challenge, particularly in resource-limited settings where maternal screening and treatment may be inadequate. The case presented herein of a preterm male neonate with a constellation of clinical findings, maculopapular rash, hepatosplenomegaly, snuffles, jaundice, micropenis, lethargy, poor feeding and an arthrogryposis-like phenotype, highlights the multisystemic impact of congenital syphilis and underscores the importance of timely diagnosis and management. Below, the authors discuss the clinical presentation, diagnostic challenges, treatment strategies and broader implications of congenital syphilis, with a focus on the unique arthrogryposis-like phenotype and neurosyphilis observed in the present case report.

The presentation of the neonate is consistent with early congenital syphilis, which typically manifests within the first 3 months of life. Classic features include maculopapular rash, hepatosplenomegaly, snuffles and jaundice, all of which were present in the case described herein (5). The high RPR titre (1:128) and positive CSF VDRL confirmed the diagnosis, aligning with CDC Category 1 criteria for proven or highly probable congenital syphilis (5). The absence of skeletal abnormalities, such as frontal bossing or Hutchinson's teeth is expected in a preterm neonate, as these features typically develop later in untreated cases (2).

The arthrogryposis-like phenotype, characterized by joint contractures, hyperextended joints and limb deformities, is a rare yet noteworthy manifestation. Arthrogryposis multiplex congenita is a heterogeneous condition defined by multiple joint contractures present at birth, often resulting from reduced fetal movement due to neuromuscular, connective tissue, or infectious etiologies (6). While congenital infections, such as cytomegalovirus (CMV) and Zika virus have been associated with arthrogryposis, reports linking congenital syphilis to this phenotype are limited (7). The mechanism likely involves syphilitic involvement of the neuromuscular system, possibly through direct spirochete invasion or inflammatory responses affecting fetal joint development. The hyperintensity of sulci on skull ultrasound and CSF abnormalities (elevated protein, low glucose and lymphocytosis) in the present case report suggest neurosyphilis, which may contribute to neuromuscular dysfunction and contractures (8).

Neurosyphilis in neonates is particularly challenging to diagnose due to the need for invasive procedures, such as lumbar puncture and the potential for false-negative CSF VDRL results (9). The positive CSF VDRL, elevated protein (258 mg/dl) and low glucose (24 mg/dl) levels in the case described herein unequivocally confirm CNS involvement, consistent with previous research reporting CSF abnormalities in 60-90% of neonates with symptomatic congenital syphilis (10). The presence of syphilitic pneumonitis, suggested by soft breath sounds and syphilitic nephritis, indicated by an overdistended urinary bladder and perinephric fluid, further illustrate the systemic nature of the disease (11).

The maternal history of a reactive RPR titre (1:16) and positive TPHA, coupled with inadequate treatment, highlights a critical gap in prenatal care. The World Health Organization (WHO) recommends universal syphilis screening in early pregnancy, with penicillin treatment for positive cases to prevent congenital transmission (11,12). Inadequate maternal treatment, defined as non-penicillin therapy, incomplete dosing, or treatment <30 days before delivery, significantly increases the risk of congenital syphilis (1). In the present case report, the high RPR titre and severe clinical manifestations of the neonate suggest *in utero* transmission due to untreated or inadequately treated maternal infection.

Neonatal evaluation is essential when maternal treatment is suboptimal. The CDC recommends evaluating all infants born to mothers with reactive serologic tests using a combination of clinical assessment, non-treponemal tests (e.g., RPR) and treponemal tests (e.g., TPHA) (1). In the present case report, the RPR titre of 1:128 in the neonate, significantly higher than the maternal titre, supports active infection, as neonatal titres are typically equal to or higher than maternal titres in congenital syphilis (9). The positive CSF VDRL further necessitated aggressive treatment, as untreated neurosyphilis can lead to long-term neurodevelopmental sequelae (6).

The neonate was treated with aqueous crystalline penicillin G (50,000 IU/kg/dose every 12 h for 7 days, then every 8 h for a total of 10 days), which aligns with the CDC and WHO guidelines for congenital syphilis with CNS involvement (5,11). Penicillin remains the only proven effective treatment, as *Treponema pallidum* lacks resistance to it (12). The dosing regimen accounts for the immature renal clearance of the preterm neonate and ensures adequate CSF penetration for neurosyphilis (13). Supportive care, including phototherapy for jaundice and supplemental oxygen for possible pneumonitis, was appropriately provided.

The arthrogryposis-like phenotype poses additional management challenges. While penicillin addresses the underlying infection, joint contractures may require physical therapy, orthopedic consultation, or surgical intervention to improve mobility (14). Long-term follow-up is critical to monitor for neurodevelopmental outcomes, given the confirmed neurosyphilis. It has been indicated that 20-30% of infants with congenital syphilis may develop neurological sequelae, including developmental delay or hearing loss, despite adequate treatment (15).

The resurgence of congenital syphilis in both high- and low-income settings reflects gaps in maternal healthcare access and adherence to screening protocols. In India, where the present case was reported, the prevalence of maternal syphilis is estimated at 1-3%, with congenital syphilis complicating up to 30% of untreated cases (16). Barriers, such as limited access to antenatal care, stigma and logistical challenges in delivering penicillin therapy contribute to persistent transmission. Strengthening antenatal screening programs, ensuring timely treatment, and improving healthcare infrastructure are essential to reducing the burden of congenital syphilis.

The arthrogryposis-like phenotype in the case described herein underscores the need for heightened awareness of atypical presentations. Clinicians should consider congenital syphilis in the differential diagnosis of neonates with joint contractures, particularly in regions with a high prevalence of syphilis. Integrating congenital syphilis screening into broader congenital infection panels (e.g., TORCH: toxoplasmosis, others, rubella, CMV and herpes) may improve diagnostic yield.

The present case report highlights several areas for future research. First, the association between congenital syphilis and arthrogryposis-like phenotypes warrants further investigation to elucidate the underlying mechanisms and prevalence. Prospective studies could explore whether syphilitic involvement of fetal neuromuscular structures contributes to contractures, potentially using advanced imaging or histopathological analysis. Second, the long-term outcomes of neonates with congenital syphilis and neurosyphilis warrant more specific characterization, particularly regarding neurodevelopmental and orthopedic sequelae. Finally, public health interventions should focus on cost-effective strategies for universal maternal screening and treatment, particularly in low-resource settings, such as India.

In conclusion, the present case report of congenital syphilis in a preterm neonate with an arthrogryposis-like phenotype and neurosyphilis illustrates the devastating multisystemic effects of untreated maternal infection. Prompt diagnosis, guided by serological and CSF findings, and treatment with penicillin are critical to improving outcomes. The rare presentation of joint contractures highlights the need for clinician awareness of atypical manifestations and underscores the importance of robust maternal screening programs. Addressing congenital syphilis requires a multifaceted approach, combining improved prenatal care, timely treatment, and long-term follow-up to mitigate sequelae.

## Figures and Tables

**Figure 1:**
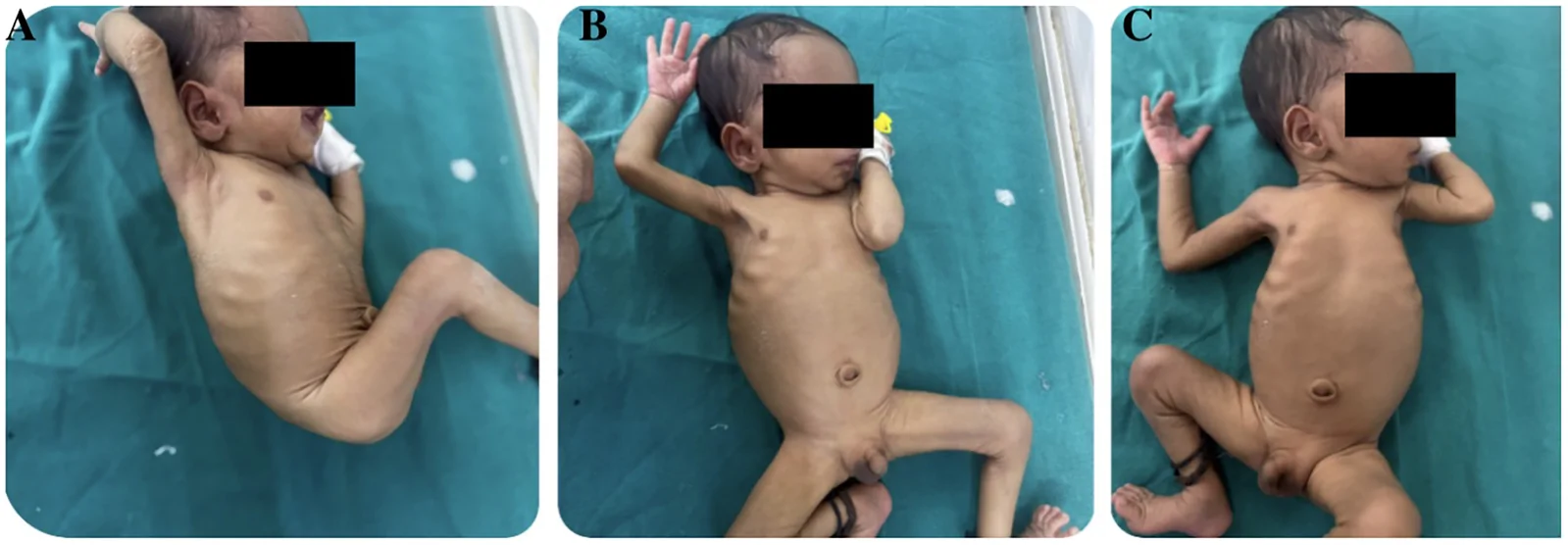
Clinical presentation of the neonate with congenital syphilis. (A) The neonate demonstrates flexion deformity of the right upper limb and extension of the left lower limb with apparent joint contractures. (B) Frontal view reveals a thin, wasted appearance with wrinkled skin, absent subcutaneous fat and bilateral lower limb deformities. (C) Lateral view illustrating hyperextended joints and fixed posture suggestive of arthrogryposis-like phenotype.

**Figure 2:**
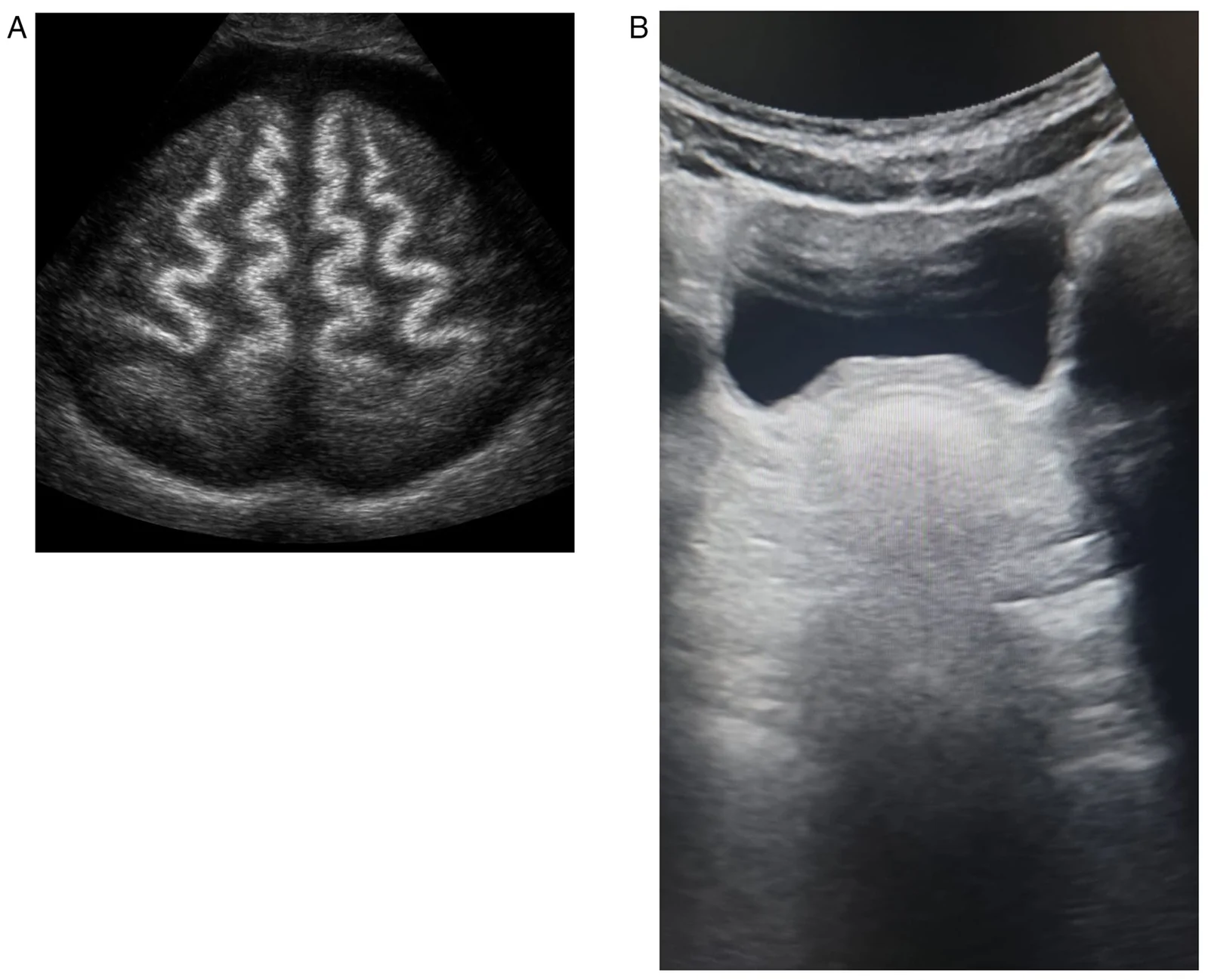
(A) Cranial (skull) ultrasound demonstrating increased echogenicity of the sulci, consistent with neurosyphilis. (B) Abdominal ultrasound demonstrating an overdistended urinary bladder with minimal perinephric fluid, suggestive of possible syphilitic nephritis.

## Data Availability

The data generated in the present study may be requested from the corresponding author.
